# Biological Activity of Phenolic Compounds in Extra Virgin Olive Oils through Their Phenolic Profile and Their Combination with Anticancer Drugs Observed in Human Cervical Carcinoma and Colon Adenocarcinoma Cells

**DOI:** 10.3390/antiox9050453

**Published:** 2020-05-24

**Authors:** Jelena Torić, Anamaria Brozovic, Mirela Baus Lončar, Cvijeta Jakobušić Brala, Ana Karković Marković, Đani Benčić, Monika Barbarić

**Affiliations:** 1 Faculty of Pharmacy and Biochemistry, University of Zagreb, A. Kovačića 1, Zagreb 10000, Croatia; jelenatoric@gmail.com (J.T.); cjakobus@pharma.hr (C.J.B.); akarkovic@pharma.hr (A.K.M.); 2 Ruđer Bošković Institute, Bijenička Cesta 54, Zagreb 10000, Croatia; Mirela.Baus.Loncar@irb.hr; 3 Faculty of Agriculture, University of Zagreb, Svetošimunska cesta 25, Zagreb 10000, Croatia; bencic@agr.hr

**Keywords:** olive oil, phenolics, biological activity, cancer cells, anticancer drugs

## Abstract

The roles of phenolics from olive oils as effective anticancer agents have been documented in various in vitro studies of different cancer cells lines, but the relationship between the phenolic profile of olive oil and its biological activity needs more elucidation. In this study, we analysed phenolic profiles of extra virgin olive oils (EVOOs) from different autochthonous cultivars from Croatia (Oblica, Bjelica, Buža, Žižolera) and investigated the biological effect of EVOO phenolic extracts (EVOO-PEs) on human cervical (HeLa) and human colon (SW48) cancer cell lines alone and in combination with cisplatin (cDDP), carboplatin (CBP), 5-fluorouracil (5-FU) and irinotecan. The quantitative evaluation of olive oil polyphenols was performed by HPLC-DAD and spectrophotometric analysis. The biological effect of EVOO-PEs alone and in combination with anticancer drugs was measured by MTT assay. Analysed EVOO-PEs differ in phenolic profile and inhibited HeLa and SW48 cells in a dose-dependent manner. Further, it is shown that EVOO-PEs (Oblica-Sea, Buža and Žižolera), in combination with anticancer drugs, increase the metabolic activity of HeLa and SW48 cells and have a protective role. These data imply careful consummation of olive oil during chemotherapy of cancer patients.

## 1. Introduction

Cancer is one of the leading causes of deaths in the world. In 2018 there were 9.6 million cases of deaths caused by cancer. According to the incident rate, lung, breast and colorectal cancer are three types of cancer ranked within the top five in terms of mortality [1,2]. As the number of cancer cases and deaths is constantly rising, cancer therapy presents one of the major medical challenges with the continuous need for improvement in the therapeutic approach. The most effective approach in the treatment of cancer is surgery combined with chemo and radiotherapy. Unfortunately, due to development of intrinsic or acquired drug resistance as well as side effects of cancer treatment, there is a growing need for more efficient anticancer agents and new strategies that could improve the success of cancer treatment. One of the possibilities is combinations of existing anticancer drugs with polyphenols.

Biological activity of single phenols, particularly hydroxytyrosol (HTyr), tyrosol (Tyr) and their derivatives oleuropein (Ole), oleacein and oleocanthal (Oc) were largely investigated on different types of cancer cells: colorectal, prostate, hepatocellular, pancreatic, cholangiocarcinoma, thyroid cancer, glioma, neuroblastoma, lung cancer, promyelocytic leukaemia, melanoma, multiple myeloma and non-melanoma skin cancer [3,4]. Anticancer properties seem to correlate with antioxidant activity of phenolic compound present in olive oil [5]. In addition, indirect antioxidant potential protects normal cells from oxidative damage [6]. These biological studies showed the beneficial effects of the most abundant phenolics in olive oil. The subject of biological activity research is also the phenolic extract of olive oil. Fabiani et al. [7] were studying the effects of olive oil phenolic extract in promyelocytic leukaemia cells (HL60) and they noticed that polyphenols protect deoxyribonucleic acid (DNA) from oxidative damage. The result of an in vitro study on colorectal cancer cell lines indicated that olive oil phenolic extract showed inhibition of certain crucial stages in the development of colorectal cancer, from initiation to promotion and metastasis [8]. Using the mouse model of colorectal cancer in the study, olive oil phenolic extract showed a significant decrease of tumour size and metastasis [9]. Another study of colon cancer cells in rats fed with EVOO analysed the effects of EVOO and its polyphenols on gene expression of endocannabinoid system components via epigenetic regulation [10]. The results of the study by Pampaloni et al. [11] indicate that EVOO-PE inhibited cell proliferation in colon cancer cells with activation of receptors, acting similar to 17β-estradiol. The anti-metastatic effect caused by polyphenols from EVOO was registered in human transitional bladder cancer cell line T24 [12]. Promising results were obtained in the study of topical application of EVOO-PE in the prevention and treatment of non-melanoma skin cancers [13]. The results of the study by De Stefanis et al. [14] indicate that EVOO extract enriched in ligstroside aglycone and oleocanthal have anti-proliferative effects on human liver cancer cell lines. Oliveras-Ferraros et al. [15] concluded that crude phenolic extracts from EVOO circumvent de novo breast cancer resistance to HER1/HER2-targeting drugs by inducing GADD45-sensed cellular stress, G2/M arrest and hyperacetylation of Histone H3. The results of studying the effect of virgin olive oil phenolic extract on HeLa cells suggest that phenolic extract has a protective effect against nuclear DNA damage in HeLa cells [16].

Moreover, the potential benefit of polyphenols in combination with different anticancer drugs was investigated showing the synergistic, additive and antagonistic effect of polyphenols [17,18]. A great number of investigations showed that single olive oil phenolics (Ole, Oc, HTyr, Ole aglycone) can reduce toxic effects or modulate the activity of anticancer drugs such as alkylating agents: cDDP, cyclophosphamide, dacarbazine (DTIC); plant alkaloids: paclitaxel; doxorubicin (DOX); monoclonal antibodies: trastuzumab (Tzb), cetuximab; hormonal agents: tamoxifen; enzyme inhibitors: lapatinib, vemurafenib; antineoplastic and immunomodulating agents: everolimus in different preclinical cancer models [4]. To the best of our knowledge, there has been only one study of olive oil phenolic extract in combination with anticancer drugs showing reduction of mitomycin C and paclitaxel anti-proliferative effect in T24 and 5637 bladder cancer cells by EVOO-PE [19].

The aim of the present study was to investigate the phenolic content of EVOO-PEs isolated from olive oils and their impact on tumour cell metabolic activity. In addition, we were interested in the biological effect of EVOO-PEs in combination with broadly used anticancer drug irinotecan, 5-FU, cDDP and CBP. Our goal was to obtain data which will additionally illuminate the use of olive oil in the diet of cancer patients during chemotherapy.

## 2. Materials and Methods

### 2.1. Reagents and Standards

Glacial acetic acid, sodium molybdate dihydrate, sodium nitrite and aluminium chloride were bought from Merck (Darmstadt, Germany). Methanol (HPLC grade), dimethyl sulfoxide (DMSO), catechin and 2,2-Diphenyl-1-Picrylhydrazyl (DPPH), benzoic acid, hydroxytyrosol, *p*-coumaric acid, homovanillyl alcohol, oleuropein, apigenin and pinoresinol were obtained from Sigma-Aldrich Chemie GmbH (Steinheim, Germany). Gallic acid, tyrosol, 3,4-dihydroxybenzoic acid, *p*-hydroxybenzoic acid, vanillic acid, syringic acid, ferulic acid, cinnamic acid, *o*-coumaric acid and Folin-Ciocalteu’s reagent were purchased from Fluka Chemie GmbH (Buchs, Switzerland), while vanillin and formic acid (99+%) were obtained from Acros Organics (Morris Plains, NJ, USA). Sodium carbonate anhydrous and sodium hydroxide were from Kemika (Zagreb, Croatia). Irinotecan was bought from Pfizer, USA, while cisplatin (cDDP) and carboplatin (CBP) were obtained from Sigma-Aldrich (St. Louis, MO, USA).

### 2.2. Olive Oil Samples

Fresh and healthy olive fruits of the variety Oblica (the island of Ugljan, Kali, Croatia) were carefully selected upon the harvest. A portion of the fruit (50.0 kg) was immediately treated with the Oliomio 350 centrifugal line to make EVOO Oblica. Another part of the olive fruit (50.0 kg) was stored in seawater before processing on the same line to get olive oil (EVOO-Sea) [20]. Another three samples were collected in Istrian Peninsula, Croatia: EVOO from variety Bjelica produced by Oleum Maris d.o.o. (Vodnjan, Croatia) and EVOO from variety Buža and Žižolera produced by family farm OPG Matteo Beluci (Vodnjan, Croatia). The samples were stored at 4 °C and kept out of light.

### 2.3. Extraction of Phenolic Compounds

The process of extraction of phenolic components from EVOO was carried out, with the use of ultrasonic-assisted liquid-liquid extraction technique (US-LLE), previously described by Jerman et al. [21] with some modification. EVOO sample (20.00 g) was dissolved in 10 mL of *n*-hexane then methanol (15 mL) was added and sonicated (3 × 10 min at 25 °C) using an ultrasonic bath (Elma Transsonic T570 HF = 320 W, Germany). Hettich centrifuge D-78532 (Tuttlingen, Germany) was used to spin the homogenates from each of the three extraction phases (15 min at 4000 rpm). Centrifuged homogenates were combined and shaken with *n*-hexane to degrease. The methanolic EVOO extracts, divided into two equal portions, were concentrated at 38 °C using a Büchi Heating Bath B-490 rotary evaporator (Büchi Labortechnik AG, Flawil, Switzerland). Two dried phenolic extracts were obtained from each EVOO sample upon successive evaporation of the methanol. One dried phenolic extract was re-dissolved in methanol for HPLC-DAD and spectrophotometric analysis and the other one was dissolved in 2 mL of DMSO (100% stock solution) for biological activity test (i.e., for the prepared solution of EVOO-PE with a dilution of 1% (*v*/*v*) 1 mL of the 100% stock solution was diluted in 100 mL flask with DMSO).

### 2.4. HPLC-DAD Analysis

Following the procedure, according to Jakobušić Brala et al. [22] and Owen et al. [23], an HPLC-DAD analysis was performed. HPLC analyses were performed in a Perkin Elmer Series 200 system (USA) with diode array detector (DAD) at 25 °C. The column used in the analysis was C18 Restek column (5 μm, 250 × 4.0 mm). The volume of the sample injected was 25 μL and the mobile phase contained acetic acid (98:2, *v*/*v*) (A) and methanol (B). The flow rate was 1 mL/min, the total duration was 45 min. The elution changes were: 95% A–5% B for 2 min, 75% A–25% B for 8 min; 60% A–40% B for 10 min, 50% A–50% B for 10 min, and 0% A–100% B until the end of the run. The UV absorption of eluates at 278 nm was monitored. Retention times (*R*_t_) of phenolic compounds were compared with those of the standards. The retention times (*R*_t_) of the standards in this system were (min): gallic acid, 9.66; hydroxytyrosol, 12.02; 3,4-dihydroxybenzoic acid, 13.74; tyrosol, 15.66; *p*-hydroxybenzoic acid, 17.68; homovanillyl alcohol, 18.13; vanillic acid, 20.18; syringic acid; 21.77, vanillin; 22.64, *p*-coumaric acid, 25.43; benzoic acid; 26.50; ferulic acid, 27.02; *o*-coumaric acid, 28.81; oleuropein, 30.20 and cinnamic acid; 35.31; pinoresinol 33.41, apigenin; 42.65. The retention time of oleacein (dialdehydic form of decarboxymethyl elenolic acid linked to hydroxytyrosol, 3,4-DHPEA-EDA) was detected according to the literature [24]. Phenolic compounds were quantified by integrating the peaks and using the appropriate 6-point calibration curves with authentic standards for all components except oleacein. Oleacein concentration was calculated from the calibration curve for oleuropein by including differences in their molecular weights. Standards stock solutions (0.1 M) were prepared in HPLC methanol. Calibration concentrations of standards were prepared in the range as expected for each compound in EVOO-PEs 0.3–80 µg/mL (3,4-dihydroxybenzoic acid, *p*-hydroxybenzoic acid, homovanillyl alcohol, vanillic acid, syringic acid; vanillin; *p*-coumaric acid, benzoic acid, ferulic acid, *o*-coumaric acid and cinnamic acid), 7–770 µg/mL (hydroxytyrosol and tyrosol) and 135–2700 µg/mL (oleuropein), 8–135 µg/mL (apigenin), 10-180 µg/mL (pinoresinol). Polytetrafluoroethylen (PTF) filters (0.20 μm/13 mm) from Macherey-Nagel GmbH & Co. KG (Düren, Germany) were used to filter EVOO-PE and standard solutions were filter sterilised prior to HPLC analysis using polytetrafluoroethylen (PTF) filters (0.20 μm/13 mm) (Macherey-Nagel GmbH & Co. KG, Düren, Germany). Concentration values of phenolic compounds were expressed as mg of phenol/kg of EVOO.

### 2.5. Total Phenols Analyses (TP)

The concentration of total phenols (TP) in methanolic EVOO-PEs was determined spectrophotometrically with Folin-Ciocalteu (FC) reagent at 725 nm according to Gutfinger [25]. The EVOO-PEs aliquots, 5 mL water and 0.25 mL FC reagent were transferred to 10 mL volumetric flasks. 1.5 mL of saturated (20%) sodium carbonate solution was added after 3 min to the reaction mixture. The solution was then diluted to 10 mL with water. The absorbance was measured at 725 nm against a methanol blank on UV-VIS spectrophotometer Hewlett Packard 8453 (Germany) two times after 30 min. Gallic acid served as a standard for preparing the calibration curve ranging 170–1020 µg/mL assay solution (*y* = 0.000833*x* + 0.0219; *R*^2^ = 0.9990; *x*: concentration of gallic acid in µg/mL; *y*: absorbance at 725 nm). The concentration of TP in extracts was expressed as mg gallic acid equivalent (GAE)/kg of EVOO.

### 2.6. o-Diphenols Analyses

According to Mateos et al. [26] the *o*-diphenol concentration in methanolic EVOO-PE was determined using sodium molybdate. Extract dilutions were prepared by mixing 0.5 mL of the extract with methanol:water (1:1, *v*/*v*). 0.5 mL of a 5% sodium molybdate solution in methanol/water (1:1, *v*/*v*) was added to 2 mL of diluted extract. The mixed content was in the dark for 15 min and the absorbance was measured spectrophotometrically at 350 nm relative to the reagent blank. The calibration curve was obtained by measuring standard gallic acid solutions, following the procedure described above. The obtained calibration curve in range of 85–1360 µg/mL assay solution was *y* = 0.000831*x* − 0.0216; *R*^2^ = 0.9984 (*x*: concentration of gallic acid in µg/mL; *y*: absorbance at 350 nm). The concentration of *o*-diphenols in extracts was expressed as mg gallic acid equivalent (GAE)/kg of EVOO.

### 2.7. Total Flavonoids Analyses (TF)

TF concentration in the methanolic EVOO-PEs was determined according to the spectrophotometric assay described by Kim [27]. Diluted EVOO-PE (1 mL) was added to a 10 mL volumetric flask together with water (4 mL), 5% solution of sodium nitrite (0.3 mL), 10% solution of aluminium chloride (0.3 mL) and incubated at room temperature for 5 min. Furthermore, in a flask with 1 M sodium hydroxide (2 mL) water up to 10 mL was added and the mixture was thoroughly mixed. The absorbance of the pink mixture was determined at 510 nm. A calibration curve was prepared with catechin in range of 20–200 µg/mL assay solution (*y* = 0.003608*x* − 0.001203; *R*^2^ = 0.9980; *x*: concentration of catechin in µg/mL; *y*: absorbance at 510 nm). The amount of TF was expressed as mg catechin equivalents (CE)/kg of EVOO.

### 2.8. Scavenging Effect Assay

The capacity of EVOO-PEs samples to scavenge the DPPH free radical was determined by the procedure described by Villaño et al. [28] with some modifications as follows. Aliquots of five different dilutions of EVOO-PE (0.1 mL) in methanol were added to 2.9 mL 7.5 × 10^−5^ M methanolic solution of DPPH radical. After shaking, the solution was in the dark for 30 min and its absorbance was measured at 517 nm. The DPPH scavenging effect was calculated as a percentage of DPPH discolouration using the equation % scavenging effect = (*A*_DPPH_ − *A*_sample_)/*A*_DPPH_ × 100, where *A*_DPPH_ is the absorbance of DPPH solution and *A*_sample_ is the absorbance of the DPPH solution with added EVOO-PE. The *EC*_50_ value was obtained from the linear regression of plotting the % of scavenging effect against the concentration of diluted EVOO-PE and expressed as the concentration of TP in EVOO-PE expressed in µg gallic acid equivalent (GAE)/mL EVOO-PE, leading to 50% reduction of the initial DPPH concentration.

### 2.9. Cell Culture and Biological Activity

Human cervical carcinoma (HeLa) cells were obtained from cell culture bank (GIBCO BRL-Invitrogen, Carlsbad, CA, USA). Human colon cancer (SW48) cells were obtained from cell culture bank (ATCC-LGC, Wesel, Germany). These cell lines were grown as a monolayer culture in Dulbecco’s modified Eagle’s medium (DMEM; Sigma-Aldrich, St. Louis, MO, USA), supplemented with 10% fetal bovine serum (FBS; Sigma-Aldrich, St. Louis, MO, USA) in a humidified atmosphere of 5% CO_2_ at 37 °C and were sub-cultured every 3–4 days. Biological activity of EVOO-PEs was determined by 3-[4-dimethylthiazole-2-yl]-2,5-diphenyltetrazolium bromide (MTT) assay [29] modified accordingly. In short, the cells were seeded into 96-well tissue culture plates. The next day, different concentrations of compounds were added to each well in quadruplicate. Upon 72 h incubation at 37 °C, the medium was aspirated, and the MTT dye (Sigma-Aldrich, St. Louis, MO, USA) was added. Three hours later, the formed formazan crystals were dissolved in DMSO, the plates were mechanically agitated for 5 min and the optical density at 545 nm was determined on a microtiter plate reader (Awareness Technology Inc., Palm City, FL, USA). The cell viability of non-treated cells and cell treated with the highest concentration of DMSO used for the highest dose of phenol extract was similar, meaning that DMSO did not influence cell viability in used experimental conditions.

### 2.10. Statistical Data Analysis

The SciPy.stats library of Python 2.7. a software (Python Software Foundation, Beaverton, OR, USA) package was used for statistical analysis. Results are shown as the mean values ± standard deviation (SD). Analysis of variance (ANOVA) tests was used for assessing significant differences among treatments. Pearson’s correlation tests were performed at the level of significance of 5% (*p <* 0.05) to obtain correlations between biological effect expressed as *IC*_65_ and polyphenol content and also between phenolic compounds with each other. A principal component analysis (PCA) was carried out on the biological activity data. The *IC*_65_ values (concentrations that induce 65% cell growth inhibition) were determined using a linear regression curve fit.

## 3. Results

### 3.1. Characteristics of Phenolic Extracts

In this study, we selected samples of five different EVOOs from cultivars: Oblica, Buža, Bjelica and Žižolera since we have expected they differ in phenolic profile. There were two samples of EVOOs prepared from olive cultivar Oblica differing in olive fruits processing. EVOO (Oblica) was prepared from the immediately processed fresh olive fruits, whereas EVOO (Oblica-Sea) was prepared from the olive fruits kept in the seawater before the process. This EVOO (Oblica-Sea) had a specific taste and different phenolic profile [20].

**Figure 1:**
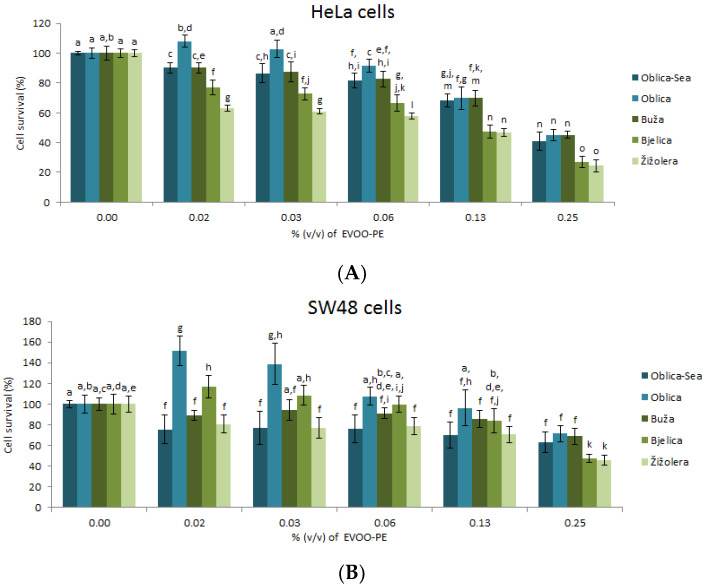
Cancer cell viability after 72 h exposure to various concentrations of EVOO-PEs derived from different cultivars (Oblica-Sea/Oblica, Buža, Bjelica and Žižolera). HeLa cells (**A**); SW48 cells (**B**). Values are the mean ± SD, *n* = 4. Means labelled by different letters are significantly different (ANOVA test, *p* ≤ 0.05).

Phenolic compounds from EVOOs were extracted using an optimised ultrasound probe assisted liquid-liquid extraction (US-LLE) method [21] and analysed using different methods. The results obtained by high-performance liquid chromatography with a diode-array detector (HPLC-DAD) analysis of EVOO-PEs under chromatographic conditions are chromatograms with peaks that correspond to different phenolics (Figure 1Sa–e). These are biomedically important HTyr, Tyr, oleacein and 10 other minor phenolic compounds: *p*-hydroxybenzoic acid, homovanillyl alcohol, vanillic acid, vanillin, *p*-coumaric acid, benzoic acid, ferulic acid, pinoresinol, cinnamic acid and apigenin. Phenolic acids like 3,4-dihydroxybenzoic, *o*-coumaric and syringic acid were not detected (ND). Oleacein, the most abundant phenol was tentatively identified by comparing its *R*_t_ in HPLC chromatograms with data found in the literature [24]. There could be some deviation in oleacein concentration, taking into account the formation of methoxy hemiacetals in methanol-water solution [30]. The values of concentrations (mg/kg EVOO) of each phenolic compound identified by HPLC-DAD analysis in the EVOO-PEs derived from different olive oil cultivars (Oblica-Sea/Oblica, Buža, Bjelica and Žižolera) are shown in Table 1. Values of total phenol (TP) concentrations are also shown in Table 1, as well as *o*-diphenols, total flavonoids (TF) of EVOO-PEs which were obtained by spectrophotometric methods.

### 3.2. Biological Effects of EVOO-PEs on Cells Survival of HeLa and SW48

Biological activity upon treatment with EVOO-PEs was evaluated by the MTT assay using in vitro experimental models: HeLa and SW48 cancer cell lines. Cells were treated with a series of dilutions that were made by diluting full strength (100%) EVOO-PE to concentrations of 0.02–0.25% (*v*/*v*) of EVOO-PE in the medium. As shown in Figure 1, all EVOO-PEs significantly inhibited HeLa and SW48 cells in a dose-dependent manner. The sensitivity of HeLa and SW48 cancer cells to EVOO-PE was expressed in terms of the concentration of extract % (*v*/*v*) required to decrease biological activity to 65% (*IC*_65_ value, Table 2). The chosen value, *IC*_65_, best describes the metabolic activity (cellular viability) of HeLa and SW48 cancer cells under the influence of all samples of EVOO-PEs. The obtained data show that EVOO-PEs from Žižolera and Bjelica had the highest biological effect on HeLa (Figure 1A) and on SW48 (Figure 1B) cell lines. The smallest effect is visible upon cell treatment with EVOO-PE isolated from Oblica (Figure 1A,B). At this moment we cannot give an explanation, based on phenolic extract content, why Žižolera and Bjelica show the best biological effect.

### 3.3. Biological Effects of EVOO-PEs in Combination with Anticancer Drugs on Cells Survival of HeLa and SW48

Different side effects of chemotherapy are well documented by Fletcher et al. [31]. In order to investigate the possible value of EVOO-PEs to the overall condition of a cancer patient, we decided to explore first the combined treatment EVOO-PEs and three types of anticancer drugs: irinotecan, 5-FU and cDDP. All the drugs are used in protocols for regular treatment of cervical and colon cancer patients. Since it is known that anticancer drugs cause different damages in cells triggering further cell death [32,33] we were interested does the pre-treatment (treatment of cancer cells with EVOO-PE overnight (ON) and then with a drug) and/or post-treatment (treatment of cancer cells first with a drug and then 6 h after with EVOO-PE) differently affect cells’ biological activity. For this purpose, 0.13% (*v*/*v*) of EVOO-PE Buža was used since this specific EVOO-PE showed medium biological effect among all EVOO-PEs tested (Figure 1A,B). The obtained data show that there were no statistical differences in HeLa cell survival upon pre- or post-treatment with EVOO-PE (Buža) in combination with tested drugs: irinotecan and 5-FU (Supplementary Materials, Figure S2a–c). However, the data obtained for the combination of EVOO-PE Buža with cDDP (Supplementary Materials, Figure S2c) showed increased viability of HeLa cells compared to cells treated only with cDDP. In order to explore this effect more closely, we decided to test other EVOO-PEs also in combination with cDDP and its derivate carboplatin (CBP).

**Figure 2:**
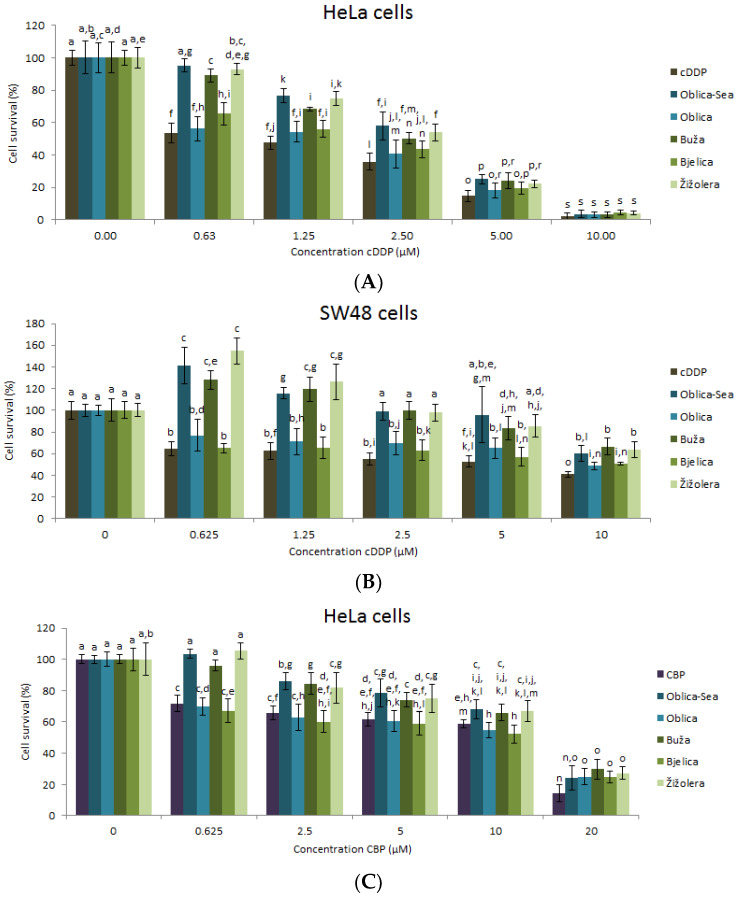
HeLa and SW48 cells viability after 72 h exposure to various doses of anticancer drugs in post-treatment (6 h after anticancer drugs) with 0.06% (*v/v*) of EVOO-PEs derived from different cultivars (Oblica-Sea/Oblica, Buža, Bjelica and Žižolera). HeLa cells with cDDP and 0.06% (*v*/*v*) of EVOO-PE (**A**). SW48 cells with cDDP and 0.06% (*v*/*v*) of EVOO-PE (**B**). HeLa cells with CBP and 0.06% (*v/v*) of EVOO-PE (**C**). Values are the mean ± SD, *n* = 4. Means labelled by different letters are significantly different (ANOVA test, *p* ≤ 0.05).

For this purpose, the post-treatment with 0.06% (*v*/*v*) dilution of EVOO-PEs was used in combination with cDDP. The biological effect of EVOO-PEs in HeLa (Figure 2A) and SW48 (Figure 2B) cells was similar. The strongest effect was visible with Oblica-Sea, Buža and Žižolera. Moreover, the similar data were obtained on HeLa cells also in combination of all EVOO-PEs with CBP (Figure 2C).

### 3.4. Statistical Analysis

Pearson correlation coefficients (r) (with the significance of *p <* 0.05 and *p <* 0.01) between biological activity expressed as *IC*_65_, antioxidant activity and polyphenol content, and also between phenolic compounds with each other are given in Table 3 and Table S1 in Supplementary Materials. As expected, the biological activity (*IC*_65_) of the EVOO-PEs on HeLa and SW48 were highly correlated to each other. Biological activities of EVOO-PEs on HeLa and SW48 were differently correlated to the phenolic compounds. In the case of HeLa *IC*_65_, there was a significant negative correlation with ferulic acid and weaker positive correlations with HTyr, homovanilyl alcohol, vanillic acid, oleacein and total phenolic alcohols and weaker negative correlation with benzoic acid. However, in the case of SW48 *IC*_65_ there was only a weak negative correlation with ferulic acid and weak positive correlations with homovanillyl alcohol and vanillic acid.

Furtherly, significant positive correlations between phenolic compounds concentration as TP and *o*-diphenols; Tyr and HTyr; oleacein and homovanillyl alcohol; cinnamic acid and Tyr; total phenolic alcohols and HTyr, Tyr, cinnamic acid and significant negative correlation as apigenin and *EC*_50_; total phenolic acid and vanillic acid were observed (Table S1 in Supplementary Materials).

**Figure 3:**
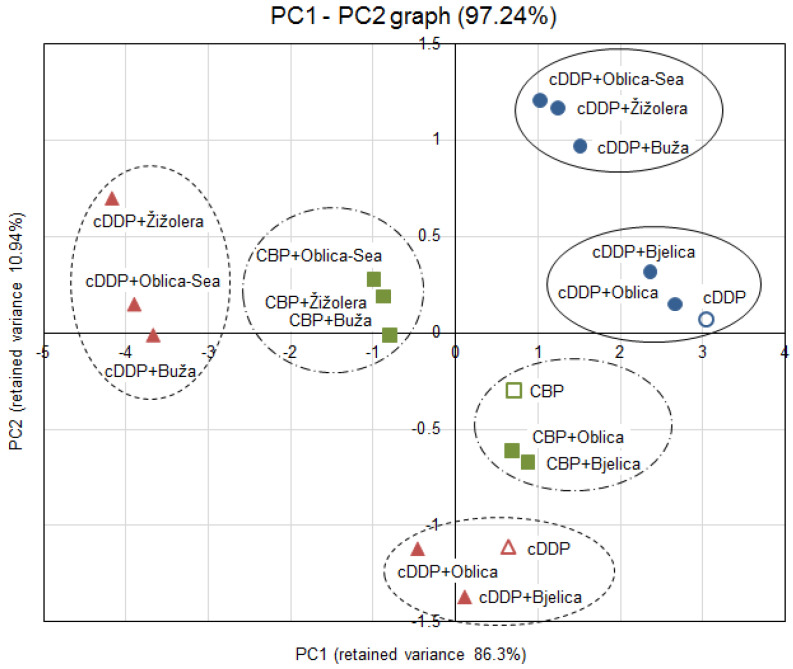
The score-plot of samples from principal competent analysis (PC1 vs. PC2). (●) HeLa cells with cDDP and 0.06% (*v/v*) of EVOO-PE. (▲) HeLa cells with cDDP and 0.06% (*v/v*) of EVOO-PE. (■) SW48 cells with cDDP and 0.06% (*v/v*) of EVOO-PE.

A principal component analysis (PCA) was carried out on biological activity data, including viability measurement of HeLa and SW48 cancer cells treated with anticancer drugs cDDP and CBP alone and in combination with five different EVOO-PEs. PCA showed that the first two principal components explained 97.2% of the total variance (Figure 3), the first one accounted for 86.3%, and the second one for 10.9%. The biplot on PC1 and PC2 shows grouping of EVOO-PE samples with Oblica-Sea, Buža and Žižolera occurring in one group, and Bjelica and Oblica in the other one, together with drug applied alone.

## 4. Discussion

The EVOO-PEs were characterised by the difference in the concentration of their main phenolic components and concentration levels of TPs, o-diphenols and TFs (Table 1). The main variations in phenolic profile were observed between EVOO Oblica (the island of Ugljan) and oils originating from the Istrian Peninsula (Buža, Bjelica and Žižolera). The difference between two samples of EVOO derived from the same cultivar Oblica is in the procedure of obtaining olive oil from its fruit. The EVOO (Oblica-Sea) is obtained through a procedure when the olive fruit is kept in the seawater and have a specific taste and different phenolic profile [20]. The EVOO (Oblica) obtained through the method of crushing olive fruits immediately after the harvest, and in comparison to other samples of EVOOs contains the highest levels of phenolic compounds, especially HTyr (21.23 ± 0.15 mg/kg EVOO), Tyr (9.26 ± 0.08 mg/kg EVOO) and oleacein (173 ± 6 mg/kg EVOO). This oil also has the highest concentration of TPs, o-diphenols and TFs but the value indicating antioxidative levels *EC*_50_ is almost the same as in the EVOOs from the Istrian Peninsula (Buža, Bjelica and Žižolera) and higher in comparison to the EVOO (Oblica-Sea) (Table 1). The EVOOs (Buža, Bjelica and Žižolera) contain similar values of all major phenolic compounds, which are lower in comparison to the EVOO (Oblica) and EVOO (Oblica-Sea). The only exception is the content of lignan pinoresinol which has the highest concentration in the EVOO (Buža) in the amount of 12.57 ± 0.06 mg/kg of EVOO. In general, high variation in phenolic content due to several factors including geographic region of olive growth, olive tree cultivar, agricultural techniques applied to cultivate olives, olive maturity and processing of the olives to oil is expected [34]. By selecting oils with different phenolic profiles, we wanted to explore the possible difference in biological activity on cancer cells. Moreover, we were interested if the use of the EVOO-PE in combination with anticancer drugs, instead of a single phenolic compound, resulted in possible synergistic effects of the observed drug and compound mixture that could be very beneficial in cancer treatments or the use of olive oil during the cancer therapy should be scheduled more properly.

According to the literature, the biological effects of olive oil phenolic extract were investigated in several types of cancer cells such as promyelocytic leukaemia cells (HL60) [7], human transitional bladder cancer cell line T24 and 5637 [12], non-melanoma skin cancers [13] and colorectal cancer cell lines [8,10,11]. In our research, the biological activity of different EVOO-PEs against HeLa cells (Figure 1A) was determined at concentrations of 0.02%, 0.03%, 0.06%, 0.13% and 0.25% (*v*/*v*) of EVOO-PE. Biological activity of EVOO-PEs on HeLa cells is in general proportional to concentration. Furthermore, the biological activity of particular EVOO-PEs observed at each concentration level specifically was different with the exception of Oblica-Sea and Buža, with Oblica showing weakest, while Bjelica and Žižolera the greatest effect. At 0.13% and 0.25% (*v*/*v*) of EVOO-PE, the difference among cultivars is less pronounced; Buža and Žižolera show a slightly higher effect on cell viability than Oblica-Sea, Oblica and Buža. In the case of SW48 cells, EVOO-PE Oblica-Sea, Buža and Žižolera have a similar effect, higher than Oblica and Bjelica at each concentration level (Figure 1B). No statistical difference was observed in the % of cell survival rate at different concentration levels of EVOO-PEs from Oblica-Sea, Buža and Žižolera cultivars with the exception of Žižolera at 0.25% (*v*/*v*) of EVOO-PE. The largest variation in the biological activity of different EVOO-PEs at the same concentration levels was observed at 0.02% (*v*/*v*). The Pearson correlation test reveals different relationship among *IC*_65_ values and phenolic compound content of different EVOO-PEs in the case of HeLa and SW48 cells. However, in order to understand which components of EVOO-PEs are responsible for the observed effect requires further investigation.

In the second part of our study, we were interested in exploring the biological effect of EVOO-PEs in combination with broadly used anticancer drugs. Chemotherapy, despite its many side effects, is still the most popular way of treating cancer and sometimes the only way. Polyphenolic compounds give hope for an improvement of chemotherapy efficacy as well as the reduction of side effects. For instance, platinum drugs are widely used in the treatment of different types of cancer; however, its application is limited because of development of drug resistance and many undesirable side effects in humans [35,36]. Nevertheless, some reports have shown that platinum drugs-induced reactive oxygen species (ROS) formation in vivo and in vitro, which is responsible for the severe side effects of cDDP therapy, including nephrotoxicity and hepatotoxicity which can be reduced by the addition of antioxidants [37]. To the best of our knowledge, there has been only one study researching the biological effect of olive oil phenolic extract in combination with different anticancer drugs. Coccia et al. [19] have investigated the biological activity of EVOO-PE with drugs paclitaxel and mitomycin C in vitro in T24 and 5637 bladder cancer cells. The authors showed that simultaneous treatment of mitomycin C and EVOO-PE reduced the drug cytotoxicity due to inhibition of ROS production. The co-treatment of T24 cells with paclitaxel and the polyphenol extract strongly increased the apoptotic cell death compared to paclitaxel alone. The authors suggested that olive oil consumption exerts health benefits and may represent a starting point for the development of new anticancer strategies. In our preliminary research, the combination of anticancer drugs (irinotecan, 5-FU, cDDP) and one of the EVOO-PEs, Buža showed no significant differences in biological effect on HeLa cell line if the cells were treated with EVOO-PE, before or after the drug was added (before DNA/after DNA damage; Supplementary Materials Figure S2). However, only the combination of EVOO-PE Buža with cDDP resulted in increased viability of HeLa cells. Following these results, cDDP was selected for further viability studies where a combination of cDDP with five different EVOO-PEs, from cultivars Bjelica, Buža, Žižolera, Oblica added 6 h after cDDP, was investigated.

Statistical analysis (PCA) was performed on biological activity data (Figure 2A–C) to analyse whether the different phenolic profiles of EVOOs (see Table 1) exert a different effect on cells survival when applied with broadly used anticancer drugs cDDP and CBP. The effect of the combination of EVOO-PE in post-treatment and drugs were examined in three different cases: the effect of EVOO-PEs with cDDP on HeLa and SW48 cells and the effect of EVOO-PEs with CBP on Hela cells. The biplot on PC1 and PC2 (Figure 3) shows that EVOO-PEs Oblica and Bjelica are in the same group with drug applied alone, while the EVOO-PEs Oblica-Sea, Buža and Žižolera form another group, in the each of three examined cases, leading to the conclusion that EVOO-PEs Oblica-Sea, Buža and Žižolera boosted the metabolic activity of cancer cells after the treatment with platinum drugs, while Oblica and Bjelica do not. There could be several reasons for this. It is known that among DNA damages cDDP and CBP can induce the formation of ROS [37,38,39]. It is known that phenolic compounds act as antioxidants and lower the effects of ROS [40]. All analysed EVOO-PEs show similar antioxidative capacity, expressed as *EC*_50_ with the exception of EVOO-PE Oblica-Sea with slightly higher capacity (Table 1). However, EVOO-PE Oblica-Sea, Buža and Žižolera show a significantly higher chemoprotective effect on cancer cells indicating there is probably some additional mechanism besides antioxidant, what could be related with a specific phenolic profile of these EVOOs. There is a possibility that EVOO-PEs interact with some proteins involved in DNA damage response by increasing their functionality. Furthermore, it is interesting to notice that EVOO Oblica-Sea obtained from olives kept in seawater before processing differs from EVOO Oblica with specific taste but also regarding different biological potential. Since EVOO (Oblica-Sea) shows a greater chemoprotective effect, it would be interesting to further investigate this issue [20]. In addition, it is important to emphasise that due to the observed EVOO-PEs chemoprotective effect on cancer cells it will be interesting to explore which phenolic compounds from olive oil could have a protective effect in the regeneration process of normal cells damaged during chemotherapy for the possible use of specific olive oil components as compounds to induce a decrease in chemotherapy side effects.

## 5. Conclusions

In this study, we have described phenolic profiles of several EVOOs from different cultivars (Oblica, Bjelica, Buža, Žižolera) of two geographic origins (island of Ugljan and Istrian Peninsula, Croatia) and investigated biological effect of EVOO-PEs on human cervical (HeLa) and human colon (SW48) cancer cell lines alone and in combination with different anticancer drugs. All EVOO-PEs have a similar biological effect on HeLa and SW48 cells but in combination with broadly used anticancer drugs the EVOO-PEs Oblica-Sea, Buža and Žižolera show the highest chemoprotective effect. These findings demonstrate the necessity of careful consummation of olive oil during chemotherapy for cancer patients. Further investigation is needed to explore the molecular mechanism of this protection and possible use of specific phenols individually for reduction of side effects of chemotherapy.

## Figures and Tables

**Table 1:** Phenolic composition and antioxidant activity of (EVOO-PEs) obtained from different cultivars.

	EVOO-PE (Cultivar)				
	Oblica-Sea	Oblica	Buža	Bjelica	Žižolera
TP (mg GAE/kg EVOO ± SD)	319 ± 48 ^A^	682 ± 8 ^B^	270 ± 8 ^A^	423 ± 38 ^A^	391 ± 34 ^A^
*o*-diphenols (mg GAE/kg EVOO ± SD)	136 ± 4 ^A^	277 ± 40 ^B^	114 ± 3 ^C^	122 ± 11 ^A,C^	128 ± 7 ^A,C^
TF (mg CE/kg EVOO ± SD)	279 ± 53 ^A^	606 ± 71 ^B^	199 ± 3 ^A^	230 ± 42 ^A^	241 ± 6 ^A^
*EC*_50_	158	191	190	208	198
Phenolic compounds (mg/kg EVOO ± SD)					
HTyr	12.73 ± 0.12 ^A^	21.23 ± 0.15 ^B^	4.07 ± 0.17 ^C^	4.85 ± 0.10 ^D^	4.48 ± 0.14 ^C,D^
3,4-dihydroxybenzoic acid	ND	ND	ND	ND	ND
Tyr	7.69 ± 0.11 ^A^	9.26 ± 0.08 ^B^	4.57 ± 0.01 ^C^	5.47 ± 0.42 ^C,D^	5.14 ± 0.12 ^D^
*p*-hydroxybenzoic acid	0.11 ± 0.10 ^A^	0.08 ±0.02 ^B^	0.15 ± 0.01 ^A^	0.09 ± 0.02 ^A,B^	0.12 ^A^ ± 0.01 ^A^
Homovanillyl alcohol	0.26 ± 0.03 ^A^	0.43 ± 0.01 ^B^	0.27 ± 0.02 ^A^	0.17 ± 0.01 ^C^	0.18 ± 0.01 ^A,C^
Vanillic acid	0.95 ± 0.01 ^A^	0.77 ± 0.02 ^B^	0.92 ± 0.01 ^A^	0.50 ± 0.01 ^C^	0.69 ± 0.01 ^B^
Syringic acid	ND	ND	ND	ND	ND
Vanillin	0.30 ± 0.01 ^A^	0.54 ± 0.01 ^B^	0.46 ± 0.01 ^C^	0.29 ± 0.01 ^A^	0.37^D^ ± 0.02 ^D^
*p*-coumaric acid	0.32 ± 0.03 ^A^	0.48 ± 0.01 ^B^	0.35 ± 0.01 ^C^	0.40 ± 0.02 ^C^	0.37 ± 0.01 ^C^
Benzoic acid	0.28 ± 0.14 ^A^	0.25 ± 0.14 ^A^	0.53 ± 0.10 ^A^	1.75 ± 0.19 ^B^	0.90 ± 0.36 ^A,B^
Ferulic acid	0.24 ± 0.04 ^A^	0.28 ± 0.03 ^A^	0.25 ± 0.01 ^A^	0.67 ± 0.07 ^B^	0.61 ± 0.04 ^B^
Oleacein	59 ± 1 ^A^	173 ± 6 ^B^	72 ± 1 ^C^	43 ± 1 ^D^	39 ± 1 ^E^
*o*-coumaric acid	ND	ND	ND	ND	ND
Pinoresinol	3.07 ± 0.04 ^A^	4.69 ± 0.01 ^B^	12.57 ± 0.06 ^C^	5.40 ± 0.33 ^B^	7.10 ± 0.16 ^D^
Cinnamic acid	0.93 ± 0.01 ^A^	1.06 ± 0.02 ^B^	0.36 ± 0.02 ^C^	0.41 ± 0.02 ^C^	0.51 ± 0.01 ^D^
Apigenin	1.36 ± 0.01 ^A^	0.99 ± 0.06 ^B^	1.01 ± 0.02 ^B^	0.87 ± 0.01 ^B^	0.85 ± 0.01 ^B^

EVOO-PE: extra virgin olive oil phenolic extract. TP: total phenols. GAE: gallic acid equivalent. SD: standard deviation. TF: total flavonoids. CE: catechin equivalent. *EC*_50_: concentration of TP in µg GAE/mL PE ± SD leading to a 50% reduction of the initial DPPH concentration. HTyr: hydroxytyrosol. Tyr: tyrosol. ND: not detected. The means within each row labelled by different capital letters A–D are significantly different (ANOVA test, *p* ≤ 0.05).

**Table 2:** Biological activity of EVOO-PEs in HeLa and SW48 cells obtained from different cultivars expressed as *IC*_65_ (in % *v/v* of EVOO-PE ± SD).

	EVOO-PE (Cultivar)				
	Oblica-Sea	Oblica	Buža	Bjelica	Žižolera
HeLa *IC*_65_ (% *v/v* of EVOO ± SD)	0.14 ± 0.02 ^A^	0.17 ± 0.02 ^A^	0.15 ± 0.02 ^A^	0.06 ± 0.01 ^B^	0.01 ± 0.01 ^C^
SW48 *IC*_65_ (% *v/v* of EVOO ± SD)	0.19 ± 0.15 ^A^	0.25 ± 0.03 ^A^	0.33 ± 0.09 ^A^	0.19 ± 0.01 ^A^	0.13 ± 0.05 ^A^

EVOO-PE: extra virgin olive oil phenolic extract. HeLa: human cervical cancer cells. SW48: human colon cancer cells. *IC*_65_: the concentration % (*v*/*v*) of EVOO-PE required to decrease biological activity to 65%. SD: standard deviation. The means within each row labelled by different capital letters A–D are significantly different (ANOVA test, *p* ≤ 0.05).

**Table 3:** Pearson correlation among the concentrations of different phenolic compounds in EVOO-PEs and their antioxidant and biological activity in HeLa and SW48 cells.

	HeLa *IC*_65_	SW48 *IC*_65_
HeLa *IC*_65_	NaN	0.881 *
SW48 *IC*_65_	0.881 *	NaN
TP	0.203	−0.067
*o*-diphenols	0.498	0.196
TF	0.494	0.176
*EC*_50_	−0.50	−0.247
HTyr	0.627	0.231
Tyr	0.558	0.12
*p*-hydroxybenzoic acid	−0.026	0.332
Homovanillyl alcohol	0.799	0.578
Vanillic acid	0.641	0.593
Vanillin	0.538	0.56
*p*-coumaric acid	0.181	0.043
Benzoic acid	−0.669	−0.483
Ferulic acid	−0.896 *	−0.782
Oleacein	0.688	0.474
Pinoresinol	0.037	0.488
Cinnamic acid	0.522	0.074
Apigenin	0.573	0.321
Total phenolic alcohol	0.617	0.213
Total phenolic acids and derivatives	−0.562	−0.594

HeLa: human cervical cancer cells. SW48: human colon cancer cells. *IC*_65_: the concentration % (*v/v*) of EVOO-PE required to decrease biological activity to 65%, TP: total phenols. TF: total flavonoids. *EC*_50_: concentration of TP in µg GAE/mL PE ± SD leading to 50% reduction of the initial DPPH concentration. Total phenolic alcohol: the sum of concentrations of HTyr, Tyr and homovanillyl alcohols. HTyr: hydroxytyrosol. Tyr: tyrosol. * *p* < 0.05.

## Supplementary Materials

The following are available online at https://www.mdpi.com/2076-3921/9/5/453/s1, Figure S1: HPLC chromatogram of EVOO-PEs at 278 nm. Oblica-Sea (A); Oblica (B); Buža (C); Bjelica (D); Žižolera (E). HTyr: hydroxytyrosol, Tyr: tyrosol. Figure S2: HeLa cells viability after 72 h exposure to various doses of anticancer drugs and pre-treatment overnight (ON) or post-treatment (6 h after anticancer drugs) with 0.13% (*v*/*v*) of EVOO-PE (Buža). Irinotecan and 0.13% (*v*/*v*) of EVOO-PE (Buža) (A). 5-fluorouracil (5-FU) and 0.13% (*v*/*v*) of EVOO-PE (Buža) (B). Cisplatin (cDDP) and 0.13% (*v*/*v*) of EVOO-PE (Buža) (C). Values are the mean ± SD, *n* = 4. Means by different letters are significantly different (ANOVA test, *p* ≤ 0.05), Table S1: Pearson correlation among the concentrations of different phenolic compounds in EVOO-PEs and their antioxidant and biological activity in HeLa and SW48 cells.
