# Supplementary material for: Biological Activity of Phenolic Compounds in Extra Virgin Olive Oils through Their Phenolic Profile and Their Combination with Anticancer Drugs Observed in Human Cervical Carcinoma and Colon Adenocarcinoma Cells
Source: Antioxidants (Basel). 2020 May 24;9(5):453. doi: 10.3390/antiox9050453 (PMC7278692; doi:10.3390/antiox9050453)
Supplement: Supplementary file 1 [file antioxidants-09-00453-s001.pdf]

Supplementary Materials

Article

Biological Activity of Phenolic Compounds in Extra Virgin Olive Oils through Their Phenolic Profile and Their Combination with Anticancer Drugs Observed in Human Cervical Carcinoma and Colon Adenocarcinoma Cells

Jelena Torić [1], Anamaria Brozovic [2*], Mirela Baus Lončar [2], Cvijeta Jakobušić Brala [1], Ana Karković Marković [1], Đani Benčić [3], Monika Barbarić [1*]

[1] University of Zagreb Faculty of Pharmacy and Biochemistry, A. Kovačića 1, Zagreb 10000, Croatia
jelenatoric@gmail.com (J.T); cjakobus@pharma.hr (C.J.B); akarkovic@pharma.hr (A.K.M.)
[2] Ruđer Bošković Institute, Bijenička cesta 54, Zagreb 10000, Croatia
Mirela.Baus.Loncar@irb.hr (M.B.L.)
3 University of Zagreb Faculty of Agriculture, Svetošimunska cesta 25, Zagreb 10000, Croatia
bencic@agr.hr (Đ.B.)
* Correspondence authors:
Anamaria Brozovic (A.B.); brozovic@irb.hr; Tel.: +385-1-4561-145; Fax, +385-1-4561-177
Monika Barbarić (M.B.); mbarbaric@pharma.hr; Tel.: +385-1-6394-472; Fax, +385-1-6394-400

Received: date; Accepted: date; Published: date

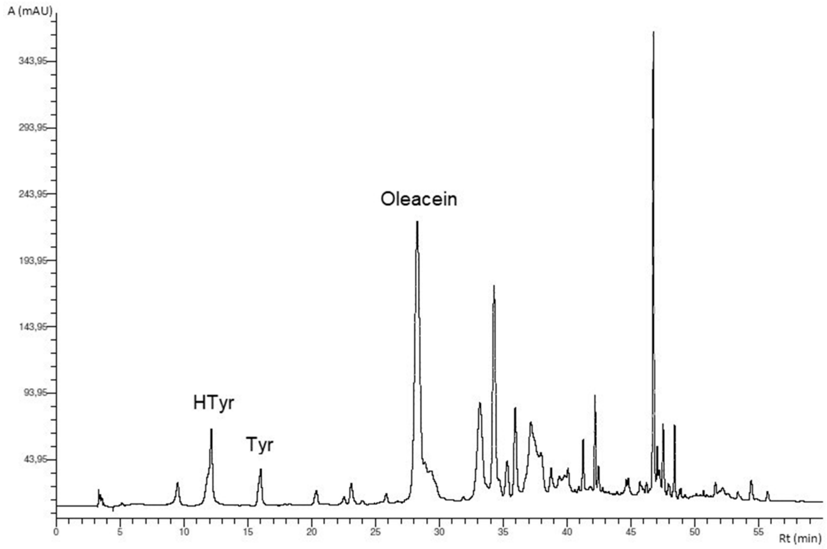

(**A**)

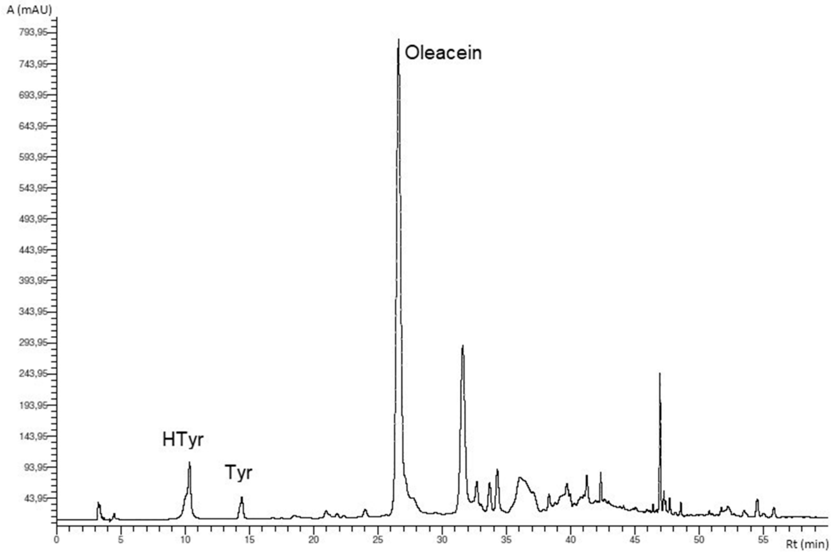
A (mAU)
793,95
743,95
693,95
643,95
593,95
543,95
493,95
443,95
393,95
343,95
293,95
243,95
193,95
143,95
93,95
43,95
Oleacein
HTyr
Tyr
0
5
10
15
20
25
30
35
40
45
50
55
Rt (min)

(**B**)

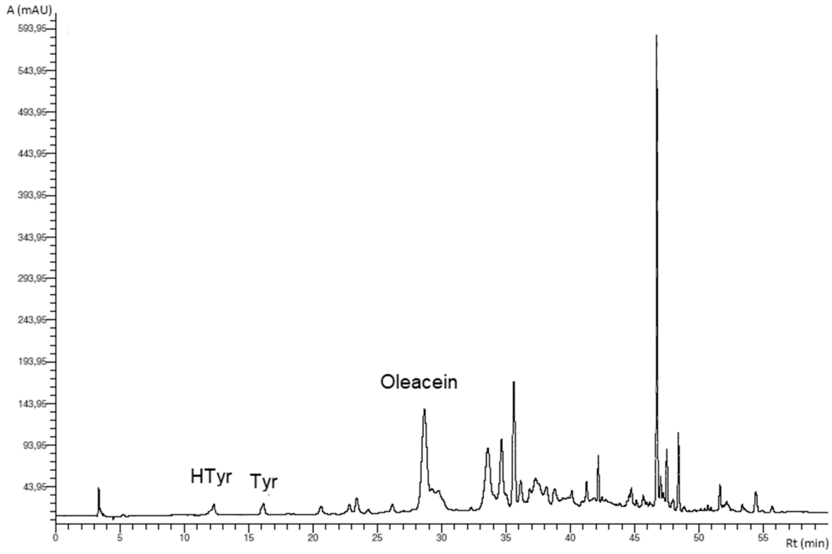
A (mAU)
593,95
543,95
493,95
443,95
393,95
343,95
293,95
243,95
193,95
143,95
93,95
43,95
Oleacein
HTyr
Tyr
0
5
10
15
20
25
30
35
40
45
50
55
Rt (min)

(**C**)

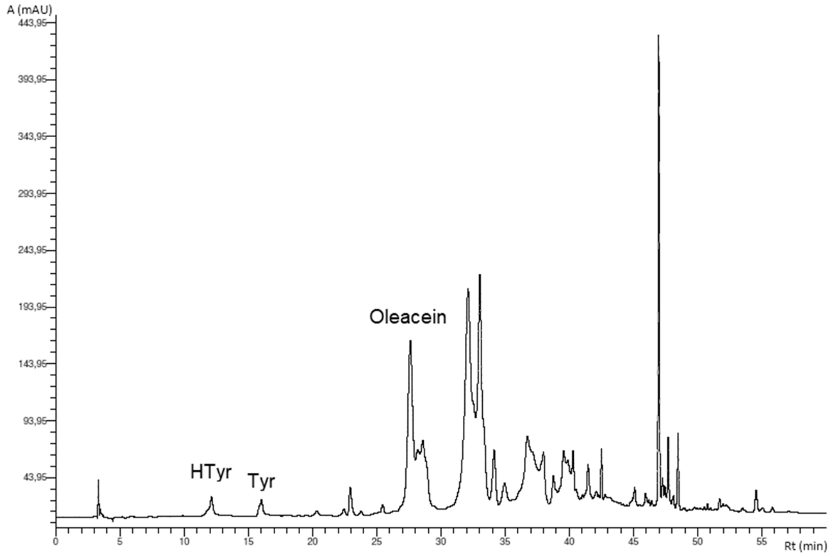
A (mAU)
443,95
393,95
343,95
293,95
243,95
193,95
143,95
93,95
43,95
Oleacein
HTyr
Tyr
0
5
10
15
20
25
30
35
40
45
50
55
Rt (min)

(**D**)

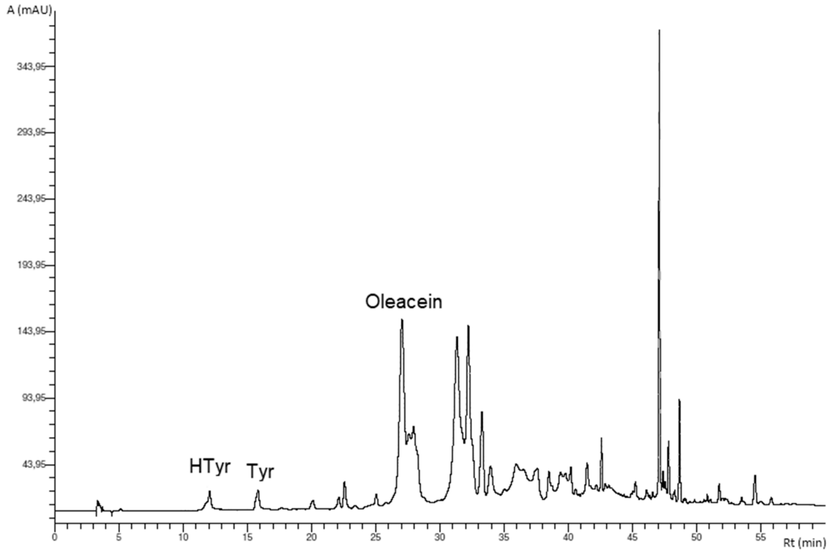

(**E**)

Figure S1. HPLC chromatogram of EVOO-PEs at 278 nm. Oblica-Sea (**A**); Oblica (**B**); Buža (**C**); Bjelica (**D**); Žižolera (**E**). HTyr: hydroxytyrosol, Tyr: tyrosol.

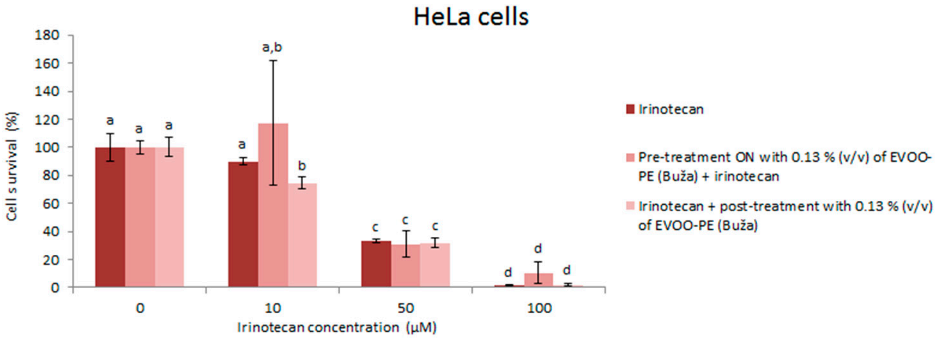

(**A**)

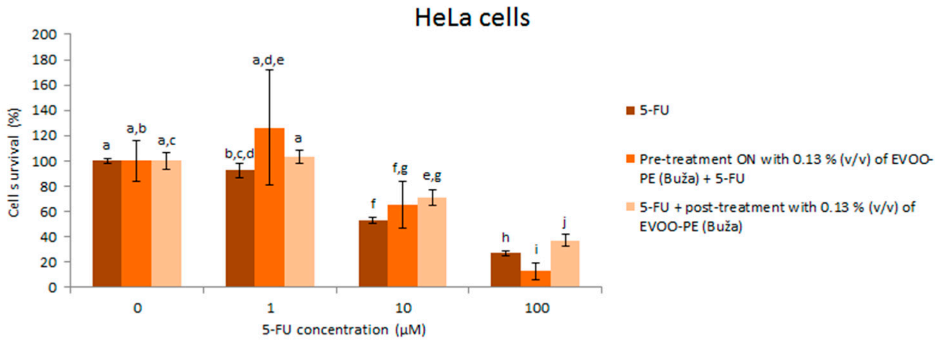

(**B**)

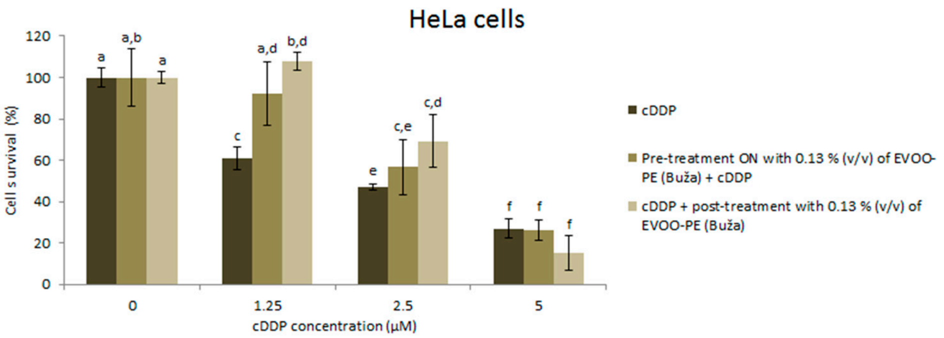

(**C**)

Figure S2. HeLa cells viability after 72 h exposure to various doses of anticancer drugs and pre-treatment overnight (ON) or post-treatment (6 h after anticancer drugs) with 0.13% (v/v) of EVOO-PE (Buža). Irinotecan and 0.13% (v/v) of EVOO-PE (Buža) (**A**). 5-fluorouracil (5-FU) and 0.13% (v/v) of EVOO-PE (Buža) (**B**). Cisplatin (cDDP) and 0.13% (v/v) of EVOO-PE (Buža) (**C**). Values are the mean ± SD, $n = 4$. Means labelled by different letters are significantly different (ANOVA test, $p \leq 0.05$).

Table S1. Pearson correlation among the concentrations of different phenolic compounds in EVOO-PEs and their antioxidant and biological activity in HeLa and SW48 cells.

	HeLa IC_{65} [a]	SW48 IC_{65} [a]	TP	*o*-diphenols	TF	EC_{50}	HTy	Tyr	*p*-hydroxy-benzoic acid	Homovanillyl alcohol	Vanillic acid
HeLa IC_{65}	NaN	0.881*	0.203	0.498	0.494	-0.504	0.627	0.558	-0.026	0.799	0.641
SW48 IC_{65}	0.881*	NaN	-0.067	0.196	0.176	-0.247	0.231	0.12	0.332	0.578	0.593
TP	0.203	-0.067	NaN	0.926*	0.926*	0.269	0.772	0.734	-0.812	0.677	-0.335
o-diphenols	0.498	0.196	0.926*	NaN	0.999**	-0.037	0.925*	0.866	-0.655	0.895*	0.042
TF	0.494	0.176	0.926*	0.999**	NaN	-0.061	0.937*	0.885*	-0.677	0.887*	0.041
EC_{50} [b]	-0.50	-0.247	0.269	-0.037	-0.061	NaN	-0.39	-0.446	-0.138	-0.269	-0.814
HTyr	0.627	0.231	0.772	0.925*	0.937*	-0.39	NaN	0.984**	-0.642	0.884*	0.26
Tyr	0.558	0.12	0.734	0.866	0.885*	-0.446	0.984**	NaN	-0.706	0.791	0.214
p-hydroxy-benzoic acid	-0.026	0.332	-0.812	-0.655	-0.677	-0.138	-0.642	-0.706	NaN	-0.324	0.528
Homovanillyl alcohol	0.799	0.578	0.677	0.895*	0.887*	-0.269	0.884*	0.791	-0.324	NaN	0.422
Vanillic acid	0.641	0.593	-0.335	0.042	0.041	-0.814	0.26	0.214	0.528	0.422	NaN
Vanillin	0.538	0.56	0.557	0.709	0.68	0.14	0.516	0.357	0.009	0.823	0.292
p-coumaric acid	0.181	0.043	0.952*	0.838	0.828	0.5	0.601	0.532	-0.703	0.615	-0.443
BenzoicaAcid	-0.669	-0.483	-0.131	-0.481	-0.48	0.717	-0.623	-0.56	-0.181	-0.731	-0.879*
Ferulic acid	-0.896*	-0.782	0.016	-0.357	-0.354	0.73	-0.539	-0.473	-0.246	-0.715	-0.909*
Oleacein	0.688	0.474	0.815	0.954*	0.945*	-0.063	0.875	0.781	-0.463	0.972**	0.202
Pinoresinol	0.037	0.488	-0.454	-0.394	-0.433	0.35	-0.577	-0.708	0.812	-0.129	0.203

Cinnamic acid	0.522	0.074	0.618	0.792	0.813	-0.588	0.952*	0.976**	-0.586	0.748	0.355
Apigenin	0.573	0.321	-0.277	0.015	0.041	-0.979**	0.383	0.448	0.08	0.257	0.758
Total phenolic alcohols	0.617	0.213	0.766	0.916*	0.929*	-0.402	0.999**	0.989**	-0.654	0.869	0.253
Total phenolic acids and derivatives	-0.562	-0.594	0.405	0.041	0.049	0.711	-0.129	-0.065	-0.658	-0.353	-0.983**

Table S1. continue

	Vanillin	*p*-coumaric acid	Benzoic acid	Ferulic acid	Oleacein	Pinoresinol	Cinnamic acid	Apigenin	Phenolic alcohols	Phenolic acids and derivatives
HeLa IC_{65}	0.538	0.181	-0.669	-0.896*	0.688	0.037	0.522	0.573	0.617	-0.562
SW48 IC_{65}	0.56	0.043	-0.483	-0.782	0.474	0.488	0.074	0.321	0.213	-0.594
TP	0.557	0.952*	-0.131	0.016	0.815	-0.454	0.618	-0.277	0.766	0.405
o-diphenols	0.709	0.838	-0.481	-0.357	0.954*	-0.394	0.792	0.015	0.916*	0.041
TF	0.68	0.828	-0.48	-0.354	0.945*	-0.433	0.813	0.041	0.929*	0.049
EC_{50}[b]	0.14	0.5	0.717	0.73	-0.063	0.35	-0.588	-0.979**	-0.402	0.711
HTyr	0.516	0.601	-0.623	-0.539	0.875	-0.577	0.952*	0.383	0.999**	-0.129
Tyr	0.357	0.532	-0.56	-0.473	0.781	-0.708	0.976**	0.448	0.989**	-0.065
p-hydroxy-benzoic Acid	0.009	-0.703	-0.181	-0.246	-0.463	0.812	-0.586	0.08	-0.654	-0.658
Homovanillyl alcohol	0.823	0.615	-0.731	-0.715	0.972**	-0.129	0.748	0.257	0.869	-0.353
Vanillic acid	0.292	-0.443	-0.879*	-0.909*	0.202	0.203	0.355	0.758	0.253	-0.983**
Vanillin	NaN	0.629	-0.568	-0.501	0.837	0.357	0.313	-0.198	0.488	-0.331
p-coumaric acid	0.629	NaN	0.023	0.101	0.782	-0.209	0.377	-0.486	0.589	0.474
Benzoic acid	-0.568	0.023	NaN	0.895*	-0.569	0.02	-0.673	-0.626	-0.614	0.837
Ferulic acid	-0.501	0.101	0.895*	NaN	-0.537	-0.103	-0.536	-0.723	-0.53	0.855
Oleacein	0.837	0.782	-0.569	-0.537	NaN	-0.162	0.7	0.059	0.86	-0.139
Pinoresinol	0.357	-0.209	0.02	-0.103	-0.162	NaN	-0.704	-0.37	-0.602	-0.363
Cinnamic acid	0.313	0.377	-0.673	-0.536	0.7	-0.704	NaN	0.557	0.959**	-0.211
Apigenin	-0.198	-0.486	-0.626	-0.723	0.059	-0.37	0.557	NaN	0.397	-0.64
Total phenolic alcohols	0.488	0.589	-0.614	-0.53	0.86	-0.602	0.959**	0.397	NaN	-0.119
Total phenolic acids and derivatives	-0.331	0.474	0.837	0.855	-0.139	-0.363	-0.211	-0.64	-0.119	NaN

HeLa: human cervical cancer cells. SW48: human colon cancer cells. IC_{65}: the concentration % (v/v) of EVOO-PE required to decrease biological activity to 65%. TP: total phenols. TF: total flavonoids. EC_{50}: concentration of TP in μg GAE/mL PE ± SD leading to 50% reduction of the initial DPPH concentration. Total phenolic alcohol: sum of concentrations of HTyr, Tyr and homovanillyl alcohols. HTyr: hydroxytyrosol. Tyr: tyrosol. NaN = not a number. * $p \leq 0.05$. ** $p \leq 0.01$%.
